# The Permanganate of Potash in the Treatment of Carbuncle

**Published:** 1867-03

**Authors:** Thad. L. Leavitt

**Affiliations:** Germantown, Pa.


### THE PERMANGANATE OF POTASH IN THE TREAMENT OF CARBUNCLE.

By THAD. L. LEAVITT, M.D., Germantown, Pa.

The beneficial effects accruing from the local use of permanganate of potash in the treatment of sloughing ulcers, phlegmonous erysipelas, and hospital gangrene, having been most thoroughly tested and proved during the last year of the war, in army hospital life, it occurred to me that its peculiar remedial qualities would alike prove successful in that most painful and distressing lesion, carbuncle, originating, as it also does, from a depressed vitality, and a morbific condition of the blood. The most satisfactory and encouraging results have been obtained in the only cases in which I have had an opportunity to employ it.

Mrs. R., *æt.* about 60 years, was visited, during the absence from town of her family physician, and found suffering terribly from a carbuncle located upon the left shoulder-blade, just above the spine of the scapula, and occupying the supra-spinous fossa. Loss of sleep, constant pain, and a naturally nervous temperament combined, induced a mental disturbance almost amounting to delirium. The tumor was in its sixth day, with all the general accompaniments, of the size of a hen's egg, tumid, tense, and shining. A free crucial incision had been made two days before, but with no relief; dense areolar tissue, puffy granulations, and sanious oozings crowded the track of the knife, with no appearance of separation or healthy action. The pulse was quick and compressible, 110 beats in the minute; countenance anxious and expressive of great pain; bowels regular. A strong solution of the permanganate of potash (ʒss. to f℥j.) was immediately applied with a brush, and a dressing saturated with it, covered with oiled silk, placed upon the shoulder. Anodynes, beef-tea, milk-punch, tincture of the chloride of iron, and quinia were administered. The same evening, the patient was again seen, and expressed herself as feeling much relieved; pulse 98, and gaining in volume and elasticity. The next morning, the dressing was removed, and already, although but twenty-five hours had elapsed, true pus had begun to form, the intense pain had subsided, and the patient, to use her own language, declared it "a miracle;" the pain had vanished, the fever was gone; she had slept well, and felt some appetite for food. A few days longer the potash was continued; the slough separated, and the wound healed in the short space of one week.

Mr. C., *æt.* 50 years, shoemaker; was visited July 30th, 1866. Had been sick three days, was found suffering intensely from a carbuncle, situated upon the abdomen just below the umbilicus, of the size of a large walnut, and involving the surrounding structures in an erysipelatous inflammation. Bowels constipated; high fever; pulse 120; heavy breath; tongue furred; anxious countenance; great restlessness and general uneasiness characterized his principal symptoms. Hop and laudanum poultices had been applied, but he had been gradually growing worse, and approaching the condition described, the tumor increasing daily, the parts becoming more dense, and at last an ichorous pus exuded from several small openings. Mild purgation, after which supporting and stimulant treatment was instituted. A slight incision was made, and the permanganate applied, as in the previous case, the dressings being removed once in twenty-four hours. This case was seen seven days successively; the 13th of August he returned to his work, the severity of the suffering having been arrested after the first application.

Mrs. A., *œt.* about 49 years, having suffered a few days from a supposed furuncle, and the pain becoming intolerable, called in medical aid. There was found upon the inner face of the left thigh, just below the nates, a well-marked, though small, carbuncle; a very slight incision was made and the potash dressing used. No constitutional treatment at all was inaugurated; in three days all signs of carbuncle had disappeared, and the line of incision was healing nicely.

The results in this case were mutually gratifying, from the fact that about six years ago the patient suffered from a series of carbuncles, appearing in succession, along the spinal column, from the back of the neck to the region of the lumbar vertebræ, and, lasting all through the winter months, her dread and fear of similar suffering were very great. The permanganate of potash has been eminently successful with me in the treatment of chronic ulcers. The following case, of many years' duration, and which had resisted all efforts, yielded to the remedial properties of this preparation.

Arthur M., tavernkeeper, *œt.* 45 years, had a chronic indurated ulcer, of sixteen years' standing, extending over the superior face of the right leg, about four inches below the tubercle of the tibia, and spreading backward on both sides to the malleoli, covering a surface of about twenty-eight square inches, deep and burrowing in some localities, and in others merely superficial; the whole leg and foot were much swollen and anasarcous, the toes merely protruding from a shapeless mass of flesh closely resembling the foot of a young elephant. An ichorous discharge of a horribly offensive character, together with filthy dressings, augmented the destruction of the surrounding parts.

The advice of an eminent surgeon had been secured a few weeks previously, to the effect that but one alternative remained, amputation; and, indeed, all appearances favored such a decision. Proper abstinence, tincture of iron, and good diet were directed. The local use of a strong solution of the permanganate of potash and judicious bandaging have already done so much for this case that, at the date of writing, the tenth application of the potash, six square inches, will more than cover the small amount of ulceration remaining, so rapid have been the healing process and the formation of firm, healthy tissue; and, in a few days more, we can confidently prognosticate a complete cure.—*Amer. Jour. Med. Sciences.*

